# Special Issue “Leishmaniasis: Pathophysiology, Diagnostics and Current and Emerging Therapeutic Approaches”

**DOI:** 10.3390/biomedicines14092041

**Published:** 2026-09-11

**Authors:** Raimunda Nonata Ribeiro Sampaio, Valdir Sabbaga Amato, Regina Maia de Souza, Daniel Holanda Barroso, João Vitor Matachon Viana

**Affiliations:** 1 University Hospital of Brasilia, University of Brasilia, Brasilia 70910-900, DF, Brazil; danielhbarroso@gmail.com; 2 Departamento de Infectologia e Medicina Tropical, Faculdade de Medicina, Universidade de São Paulo, São Paulo 05403-000, SP, Brazil; valdiramato@usp.br (V.S.A.); joao.matachon@hc.fm.usp.br (J.V.M.V.); 3 Laboratório de Parasitologia, Instituto de Medicina Tropical, Faculdade de Medicina, Universidade de São Paulo, São Paulo 05403-000, SP, Brazil; regina.maia@hc.fm.usp.br

## 1. Introduction

Leishmaniasis remains a major neglected tropical disease and a persistent challenge for biomedical research. Despite recent advances in the biological understanding of *Leishmania*, the clinical management of the disease continues to be constrained by diagnostic limitations, heterogeneous clinical manifestations and scarce therapeutic options—which carry the potential for toxicity and antimicrobial resistance. Notably, approximately one billion people live in areas at risk of contracting leishmaniasis, a disease that disproportionately affects populations with greater socioeconomic vulnerability [1].

These challenges have led to endured research into the pathophysiology of *Leishmania*, new diagnostic methods, and alternative therapeutic strategies. This Special Issue brings five studies together that address different aspects of these challenges. They include the role of Nrf2 signaling, the use of artificial intelligence (AI) to identify cutaneous lesions, the evaluation of new selenium-containing compounds, a nanoparticle-based drug delivery system, and the detection of *Leishmania braziliensis* DNA after treatment [2,3,4,5,6].

Although these studies use different approaches, they share a common goal: improving our ability to understand, diagnose, and treat leishmaniasis. Taken together, they also show that progress in the field will require closer connections between basic research and clinical practice.

## 2. Host–Parasite Interactions and Oxidative Stress

The review by Saha et al. focuses on nuclear factor erythroid 2-related factor 2 (Nrf2), a transcription factor that regulates cellular responses to oxidative stress and inflammation [2]. Oxidative stress is an important part of the host response to *Leishmania*. Reactive oxygen species can contribute to parasite control inside macrophages, but excessive oxidative stress can also cause tissue damage. At the same time, *Leishmania* can alter macrophage signaling and antioxidant pathways, which may help the parasite survive inside host cells.

Nrf2 is therefore an important pathway to study. The review summarizes evidence that Nrf2-related signaling can influence parasite survival and intracellular growth [2]. It also discusses experimental studies in which changes in such pathways affected oxidative stress, iron metabolism, and parasite burden. However, the role of Nrf2 is not straightforward. Its effects may depend on the *Leishmania* species, the infected cell, and the stage of infection. Indeed, the review emphasizes that Nrf2 may have effects that benefit both the host and the parasite, highlighting the complexity of this pathway during infection.

For this reason, Nrf2 should be considered a potential therapeutic target rather than an established treatment strategy. Future studies should determine when Nrf2 activation helps the host control infection and when it may well favor parasite survival. This distinction will be important for the development of treatments that modify the host response without producing unintended effects.

## 3. Improving the Diagnosis of Cutaneous Leishmaniasis

The diagnosis of cutaneous leishmaniasis (CL) remains challenging, especially because lesions can resemble several other infectious, inflammatory, and neoplastic skin diseases. This is particularly important in areas where access to laboratory testing is limited.

Leal et al. investigated whether AI could help identify CL from photographs of skin lesions [3]. The study included 2458 images, with up to 10 images obtained from each lesion, and used an AlexNet deep-learning model to distinguish CL from 26 other dermatoses. Across three simulations, the model achieved a mean accuracy of 95.04% (95% CI, 93.81–96.04%) [3].

These results are encouraging, but they need to be interpreted in the context of the study design. The images were obtained from patients treated at the University Hospital of Brasilia, Brazil, between 2015 and 2022. Several factors may affect the performance of an image-based model, including image quality, lighting, focus, and lesion characteristics. In addition, the use of multiple images from the same lesion limits the independence of the dataset.

The next step is therefore external and prospective validation. AI models should be tested in different endemic regions, patient populations, and clinical settings. They should also be evaluated with images obtained under less controlled conditions, since this is closer to their potential use in routine care.

AI is unlikely to replace parasitological or molecular testing. Its main value may be as an additional tool that assists clinicians and general practitioners recognize suspicious lesions and identify patients who require confirmatory testing.

Other diagnostic approaches are also being investigated. Recent work has identified new candidate antigens for the serological diagnosis of CL [7]. These approaches may eventually complement existing methods and improve diagnostic accuracy worldwide.

## 4. New Compounds and New Approaches to Drug Delivery

The limited number of effective antileishmanial drugs remains a major barrier to better treatment. Current medicines can be effective, but toxicity, route of administration, treatment duration, and variable responses continue to create important limitations to its use.

Fermiano et al. evaluated ten selenium-containing heteroaryl compounds against *L. amazonensis* [4]. Among the compounds tested, MRK-106 and MRK-108 showed the highest activity against promastigotes, with IC_50_ values of 3.97 and 4.23 μM, respectively. MRK-107 and MRK-113 also showed activity against intracellular amastigotes, with IC_50_ values of 18.31 and 15.93 μM, respectively. Remarkably, the compounds performed differently depending on the parasite stage, with some showing greater activity or selectivity against promastigotes and others against intracellular amastigotes.

These results highlight an important point in antileishmanial drug development: activity against promastigotes does not necessarily predict activity against intracellular parasites. Since amastigotes are the form observed inside host cells, activity against this stage is particularly relevant when selecting compounds for further development.

A useful drug candidate must combine antileishmanial activity with low toxicity to host cells. Further studies should therefore examine the mechanisms of action, pharmacological properties, and efficacy and safety of these compounds in vivo.

Galue-Parra et al. explored a different strategy [5]. They developed silica nanoparticles functionalized with sialic acid and colchicine to improve the delivery of colchicine to target *L. braziliensis* parasites. The formulation was designed to promote parasite interaction with the nanostructure and improve the selectivity of the treatment.

The results were promising in vitro, with a selectivity index increased from 20.32 for colchicine alone to 57.89 for the nanoparticle formulation against extracellular parasites. For intracellular amastigotes, the selectivity index increased from 42.06 to 88.88 [5].

These findings suggest that drug delivery can be as important as the choice of the active compound. Yet, as the study remains preclinical, the formulation needs to be evaluated in vivo, including its safety, pharmacokinetics, tissue distribution, and therapeutic efficacy.

The two experiments therefore represent complementary approaches. One requests new compounds with antileishmanial activity, while the other requests to improve the delivery of an existing drug. Both strategies might help expand the limited therapeutic options available for leishmaniasis.

## 5. Parasite Persistence After Treatment

The study by Lopes et al. provides insights into parasite persistence after treatment [6]. The authors evaluated 72 patients with leishmaniasis and detected *L. braziliensis* DNA in nasal mucosal samples from five patients immediately after treatment. Among these five patients, 60% had mucosal lesions before treatment, compared with 13.4% of patients who were negative for *L. braziliensis* DNA after treatment.

The finding is relevant because mucosal disease may develop long after the initial cutaneous infection. Some patients may have few symptoms during the early stages of mucosal involvement. A marker associated with mucosal involvement could therefore help identify patients who require closer and/or longer follow-up.

Nevertheless, PCR positivity should not be interpreted as evidence of active infection. Detection of parasite DNA does not demonstrate that viable parasites are present. Prominently, the study cannot establish whether PCR positivity after treatment predicts future mucosal disease or relapse, because the patients were not followed for a sufficiently long period [6].

For these reasons, the research should be perceived as a starting point for further investigation. Longitudinal studies are needed to determine whether molecular detection after treatment is associated with later clinical outcomes.

This issue is part of a broader challenge in leishmaniasis: clinical cure may not provide a complete picture of the biological status of infection. The persistence of parasite-derived material after clinical healing also raises questions about whether clinical assessment alone is sufficient to define treatment success [8].

## 6. From Experimental Research to Clinical Practice

The studies in this Special Issue are part of a broader change in leishmaniasis research. New technologies are being developed, but their value will ultimately depend on whether they can improve patient care.

Recent clinical data illustrate the importance of studying existing treatments in real-world settings. In a prospective study of 127 patients with localized CL in Brazil, intralesional meglumine antimoniate achieved an effectiveness of 69.3% in the intention-to-treat analysis and 76.8% in the per-protocol analysis after the initial regimen. Effectiveness increased to 85.6% after an additional treatment cycle [9].

The study also used mobile health tools to support patient follow-up. This approach is particularly relevant in endemic areas where patients may live far from specialized centers. Digital tools may help maintain follow-up while reducing the need for repeated travel.

At the same time, new oral treatments are being evaluated. LXE408 is being studied in a randomized, multicenter Phase II trial in patients with localized CL in Brazil and Panama. The protocol compares two LXE408 regimens with oral miltefosine and includes follow-up through Day 180 [10].

The development of new oral drugs is important because the route of administration remains a practical limitation of most current therapies. Nevertheless, their clinical value will depend not only on short-term efficacy but also on sustained cure, relapse prevention, safety, and the potential need for additional treatment.

Recent guidelines have emphasized that treatment should be selected according to the *Leishmania* species, clinical presentation, lesion characteristics, and patient factors [1]. This supports a more individualized approach to CL treatment.

## 7. Outlook

The five studies included in this Special Issue address different sections of the identical problem. The work on Nrf2 may improve our understanding of how the host response affects parasite survival. AI might assist clinicians and general practitioners recognize CL among other dermatological diseases. New compounds may provide starting points for drug development, while nanoparticle systems may improve the delivery of existing drugs. Molecular detection after treatment may help identify patients who require closer follow-up. The real challenge now is to determine which of these findings can be translated into clinical practice.

Access should also remain part of this discussion. A diagnostic test that is too expensive or difficult to perform will have limited value in endemic areas. The same is true for treatments that require resources unavailable in local health services. New technologies should therefore be evaluated not only for their biological performance but also for their cost, availability, and feasibility.

The main message of this Special Issue is that progress in leishmaniasis will require advances at several levels. We need to better understand the interaction between the parasite and the host, improve diagnosis, develop safer and more effective treatments, and improve the way patients are followed after therapy.

The next step is to connect these advances. Better diagnosis should help guide treatment. Better treatments should be followed by better methods to assess cure. A better understanding of parasites and host biology should also help identify new targets for diagnosis and therapy.

Together, these approaches may move leishmaniasis care beyond the treatment of the visible skin lesion and toward a more complete understanding of infection, treatment response, and long-term disease.

## Abbreviations

The following abbreviations are used in this manuscript:

AI	Artificial Intelligence
CI	Confidence Interval
CL	Cutaneous Leishmaniasis
DNA	Deoxyribonucleic Acid
IC_50_	Half-Maximal Inhibitory Concentration
Nrf2	Nuclear Factor Erythroid 2-Related Factor 2
PCR	Polymerase Chain Reaction
